# An Analytical Calculation of Frictional and Bending Moments at the Head-Neck Interface of Hip Joint Implants during Different Physiological Activities

**DOI:** 10.3390/ma9120982

**Published:** 2016-12-05

**Authors:** Hamidreza Farhoudi, Reza H. Oskouei, Ali A. Pasha Zanoosi, Claire F. Jones, Mark Taylor

**Affiliations:** 1 Medical Device Research Institute, Flinders University, Adelaide 5042, Australia; hamidreza.farhoudi@flinders.edu.au (H.F.); mark.taylor@flinders.edu.au (M.T.); 2 Faculty of Industrial & Mechanical Engineering, Qazvin Branch, Islamic Azad University, Qazvin 15195-34199, Iran; aliakbar.pasha@qiau.ac.ir; 3 Adelaide Centre for Spinal Research, Adelaide 5000, Australia; claire.jones@adelaide.edu.au; 4 School of Mechanical Engineering, University of Adelaide, Adelaide 5005, Australia; 5 Centre for Orthopaedic and Trauma Research, School of Medicine, University of Adelaide, Adelaide 5005, Australia

**Keywords:** total hip replacement, head-cup interface, frictional moment, physical activities

## Abstract

This study predicts the frictional moments at the head-cup interface and frictional torques and bending moments acting on the head-neck interface of a modular total hip replacement across a range of activities of daily living. The predicted moment and torque profiles are based on the kinematics of four patients and the implant characteristics of a metal-on-metal implant. Depending on the body weight and type of activity, the moments and torques had significant variations in both magnitude and direction over the activity cycles. For the nine investigated activities, the maximum magnitude of the frictional moment ranged from 2.6 to 7.1 Nm. The maximum magnitude of the torque acting on the head-neck interface ranged from 2.3 to 5.7 Nm. The bending moment acting on the head-neck interface varied from 7 to 21.6 Nm. One-leg-standing had the widest range of frictional torque on the head-neck interface (11 Nm) while normal walking had the smallest range (6.1 Nm). The widest range, together with the maximum magnitude of torque, bending moment, and frictional moment, occurred during one-leg-standing of the lightest patient. Most of the simulated activities resulted in frictional torques that were near the previously reported oxide layer depassivation threshold torque. The predicted bending moments were also found at a level believed to contribute to the oxide layer depassivation. The calculated magnitudes and directions of the moments, applied directly to the head-neck taper junction, provide realistic mechanical loading data for in vitro and computational studies on the mechanical behaviour and multi-axial fretting at the head-neck interface.

## 1. Introduction

The mechanical environment of hip joint implants is complex and not well understood. Mechanical loads can contribute to implant failure via different mechanisms such as fretting-corrosion at the head-neck junction [1,2], loosening of the acetabular cup and femoral stem interface [3], fracture due to fatigue [4], and wear of hard-on-soft or hard-on-hard bearing couples [5,6]. The mechanical loads resulting from activities of daily living induce contact forces, frictional moments, and bending moments on the implant and its interfaces [7,8]. Head-cup contact forces are induced by body weight and muscle contraction forces, bending moments result from the offset of contact forces [9], and frictional moments are induced by the rotation of the head inside the cup (bearing couple) in the presence of friction [10,11]. Hip implant failure mechanisms such as fretting-corrosion at the head-neck interface [12], especially in larger diameter metal-on-metal bearings, are thought to be influenced by frictional moments. Fretting wear is driven by relative micro-motion at the head-neck interface, which is significantly influenced by the mechanical loading environment [13]. 

To determine head-cup contact forces, in vivo telemetry measurements have been conducted. English and Kilvington [14] employed a prosthesis equipped with strain gauges to determine load magnitude, but not orientation. Bergmann et al. [15] measured hip joint force magnitudes, orientations, and moments about the femoral stem axis during walking and running gaits, and various daily activities [9] via telemetry with an instrumented hip implant; these data are available on the Orthoload website (orthoload.com). However, these techniques are extremely costly and complicated to develop for different types of implants.

A review of the literature confirms that the contact forces and kinematics of hip joint implants are well-understood [9,15,16]. However, further research is needed to determine the frictional moments induced by the relative motion between the head and cup in the presence of friction and bending moments due to the offset of changing contact forces. As a matter of significance, the frictional moments can contribute to the failure of total hip replacements (THR). For instance, a frictional torque of 3.92 ± 0.97 Nm can cause depassivation of the oxide layer and thus initiate fretting-corrosion at a Ti–6Al–4V neck and Co–28Cr–6Mo head interface assembled with a 4.5 kN force. This depassivation torque increases to 7.23 ± 0.55 Nm for a 6 kN assembled implant [17]. The depassivation torque is normally obtained empirically by monitoring the potential or current results of the oxidation and reduction [17]. The frictional moment can also contribute significantly to polyethylene wear and aseptic loosening, which is the cause of 20%–40% of retrieval surgeries [18]. The heat induced by the frictional moments can also increase the local temperature to 43.1 °C, at which bone tissue may be damaged [19]. Panagiotidou et al. [8] reported that an increase in the bending moment (by increasing the offset from 0 to 7 mm and then to 14 mm), increases the oxide layer depassivation current for CoCr/CoCr and ceramic/CoCr head-neck combinations. This indicates that the fretting corrosion behaviour of the head-neck materials can be influenced by the bending moments induced by the offset.

In order to develop more realistic in vitro experiments and computational models, a full understanding of the frictional and bending moments in terms of both magnitude and direction is required. Most finite element (FE) studies have used simplified loading for simulations which can significantly influence the validity of the outcomes. Examples include studies on the fixation of hip resurfacing arthroplasty [20], stress shielding of the stem material [21] and wear-fatigue in modular hip implants [22]. 

To determine the frictional moments, Damn et al. [7] used an instrumented implant to measure forces and frictional moments, and derived friction coefficients during walking for a 32 mm ceramic-on-polyethylene bearing couple in vivo. However, frictional moments measured for a specific head-cup bearing couple configuration cannot be generalized to other bearing with different geometries and material combinations. Frictional moments are dependent on the friction coefficient of the bearing couple, the lubrication regime, the bearing clearance, the size of the implant, and the geometry and material combination of the bearing couple [10,11,23]. Thus, the state space including all material combinations, geometries (head-cup sizes, neck offset, and version/anti-version), and daily activities is too large to be studied by in vivo experimental methods.

The authors, in their previous work [23], introduced and detailed a new methodology to analytically determine frictional moments at the head-cup bearing couple of a total hip replacement. The theoretical frictional moment results were successfully verified against experimental results for a simplified gait cycle reported by Bishop et al. [11]. However, mechanical loading data is needed for common physical daily activities of patients so that realistic loading situations can be determined for a better understanding of failures. Moreover, the existing literature confirms that there is little information about the bending moments at the head-neck taper junction; hence, these moments should also be determined and quantified. 

In this work, bending moments, frictional moments at the head-cup interface, and frictional torques acting on the head-neck junction are determined for metal-on-metal bearings during nine activities of daily living. This study provides useful data on the magnitude and direction of frictional and bending moments generated by different head sizes and body weights. These mechanical loads of various physical activities are essential for a quantitative understanding of the mechanical behaviour and failure of the taper junction in vivo without which computational and in vitro testing methods cannot be developed. 

## 2. Methods

An analytical method was developed to find the bending moment vector ($\vec{M_{b}}$), frictional moment vector ($\vec{M_{f}}$), and frictional torque vector about the head-neck interface ($\vec{M_{n}}$) under contact forces during nine different activity cycles. The friction coefficient of the bearing couple is a function of contact forces, kinematics, and material properties which in the presence of a lubricant (i.e., synovial fluid of a hip joint) results in different regimes of lubrication [24]. Currently, the elasto-hydrodynamic lubrication (EHL) theory seems to be the most comprehensive analytical model to simulate lubrication regimes and frictional moments at the bearing interface of hip joint implants. In this method, the elastic deformation of the contact area, the equilibrium equations, and the generalized Reynolds equations of the lubricant film are solved simultaneously. To decrease the number of unknowns to the number of equations (determined system), EHL uses the contact force only in the vertical direction and the rotational motion in the flexion-extension direction (2 degree of freedom, DOF), and assumes that the other two components of the force and rotational motion are zero [25,26]. This simplification cannot be expanded to a quantitative 6 DOF study as the system is indeterminant. Assuming a constant friction coefficient for hard-on-hard couples (combinations of metals and/or ceramics) in a 6 DOF problem is justified for the purpose of finding maximum values, changes of direction, and an overall trend of variation in frictional moment [23]. This assumption was successfully validated in the previous work [23], where good agreement was found between the experimental and analytical frictional moment results for hard-on-hard bearing couples, mainly as a result of the direct relationship between the contact force and friction coefficient in these couples.

**Figure 1:**
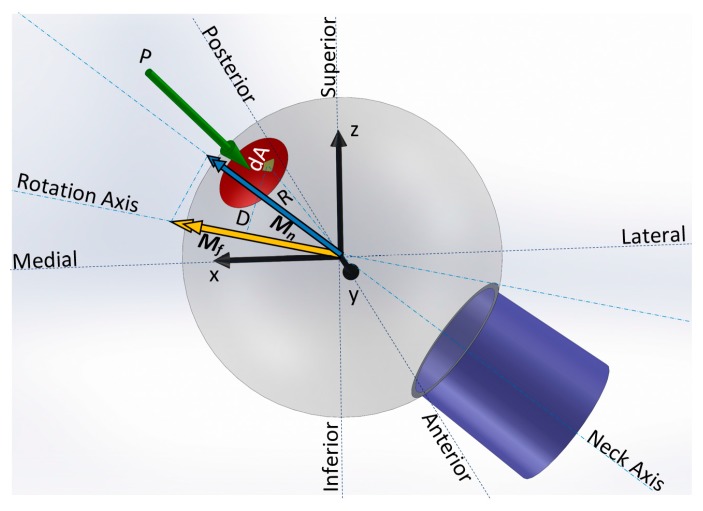
Head-neck taper junction of a left hip implant and femur coordinate system. The contact force *P* distributed over the Hertz contact area induces a tangential frictional force during the relative motion of the head and cup. The tangential force and lever arm *D* produce the frictional moment *M*_f_ and its projection onto the neck direction which is the frictional torque *M*_n_.

The method determines the bearing frictional moment (*M*_f_) using the normal Hertz contact pressure (p), friction coefficient $\left(\mu_{k}\right)$, and perpendicular distance to the rotation vector (lever arm D), as shown in Figure 1. Moment vector $\vec{M_{f}}$ is produced about a rotation axis that is the direction of rotation of the head with respect to the cup, characterised by rotation vector $\vec{V_{r}}$.

Each activity cycle was sampled for n steps (n=200). Instantaneous $\vec{V_{r}}$ was determined from the available kinematics [9], by means of Euler’s rotation theorem and the direction cosine rotation matrix. The direction of the contact force vector ($\vec{P}$) is presented in the femur coordinate system in other studies [9]. Using $\vec{P}$ and $\vec{V_{r}}$ for each instant of an activity, the normal Hertz contact pressure (p) and its lever arm from the rotation axis (D) were obtained. The frictional moment was calculated such that the parameters were transformed to a spherical coordinate system with its z axis aligned with the instantaneous force direction (Equation (1)). The calculated frictional moment was then projected onto the instantaneous axis of the neck to obtain the torque acting on the neck within the junction (*M*_n_). A detailed description of the analytical approach for formulating frictional moment (*M*_f_) has been described previously [23].

(1) $$M_{f}=\int D\cdot \mu_{k}\cdot p\cdot dA$$

To calculate the bending moments induced at the head-neck interface, the instantaneous distance vector of the contact force from the clamping region of the head and neck was formed from the available kinematics of the head with respect to the cup. The offset of the contact region of the head and neck from the force was assumed to be 10 mm which is near the common geometry of a neutral 12/14 taper design. The cross product of the instantaneous distance vector and the corresponding contact force (the bending moment induced at the head-neck clamping interface) was determined for the entire activity cycle. 

**Figure 2:**
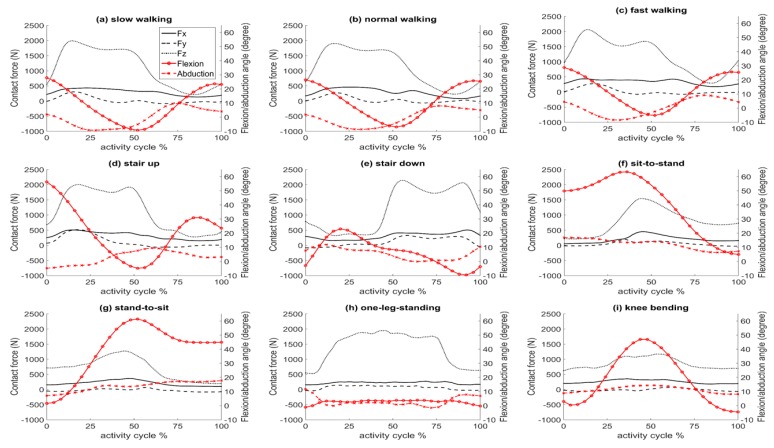
Contact forces in the *x*, *y*, and *z* axes along with flexion and abduction angles for one cycle of each activity for the average patient, data from Hip98 and Orthoload [16]: (**a**) slow walking; (**b**) normal walking; (**c**) fast walking; (**d**) stair up; (**e**) stair down; (**f**) sit-to-stand; (**g**) stand-to-sit; (**h**) one leg standing and (**i**) knee bending.

In this manner, the bending moments, frictional moments, and torque at the head-neck interface were determined for one cycle of nine activities taken from the Orthoload website: slow, normal, and fast walking, stair up and stair down (ipsilateral foot contacting ground), sit-to-stand and stand-to-sit (two legged), one-leg-standing (ipsilateral), and knee bending (two legged). Patient dynamics data were obtained from the Hip98 software available in the Orthoload database [9] for three cases: lightest patient (702 N), heaviest patient (980 N), and an average patient (836–920 N, as defined in Hip98). Dynamics of the average patient are illustrated in Figure 2. For all cases, the investigations were carried out for a CoCr/CoCr bearing couple with a nominal diameter of 46 mm and clearance of 54 μm (cup diameter: 46.018 mm and head diameter: 45.964 mm) and a friction coefficient of 0.12 [11]. This friction coefficient corresponded to the maximum contact load instance. The friction coefficient is a function of lubrication regime, material combination, and bearing geometry, and varies throughout the activity cycle. An analysis previously conducted on different bearing couples (metal-on-metal, metal-on-polyethylene, metal-on-ceramic, and ceramic-on-ceramic) showed that, for metal-on-metal bearing couples, assuming a constant friction coefficient (corresponding to the maximum contact force) was in good agreement with in vitro experimental results for a simplified activity cycle [23]. The accuracy of using a constant friction coefficient for determining frictional moments in hard-on-hard bearing couples has been justified and presented previously [23]. To determine the effect of implant head diameter on the frictional moments and torque at the head-neck interface, the normal walking activity of the average patient was simulated for three bearing diameters (28, 46, and 70 mm) representative of commonly used heads in total hip replacement and hip resurfacing.

## 3. Results

**Figure 3:**
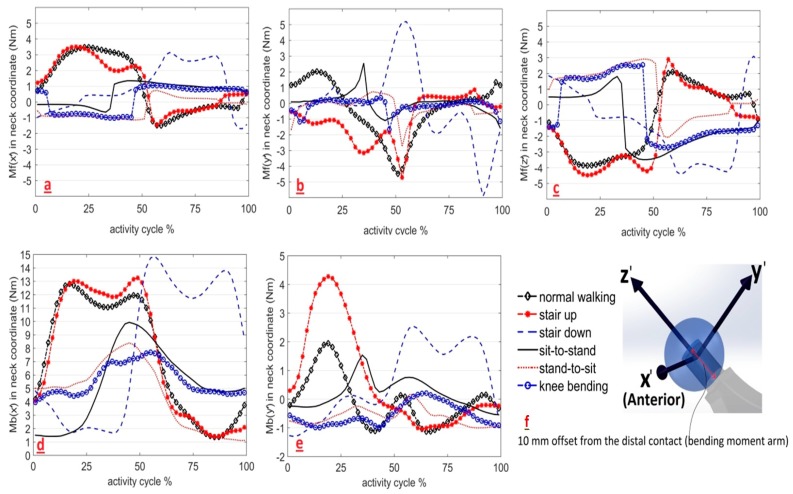
Components of frictional moments and bending moments for six daily activities in the neck coordinate system (*x*’*y*’*z*’): (**a**) Frictional moments about the *x*’ axis; (**b**) Frictional moments about the *y*’ axis; (**c**) Frictional moments about the *z*’ axis; (**d**) Bending moments about the *x*’ axis; (**e**) Bending moments about the *y*’ axis; and (**f**) Coordinate system definition.

Frictional and bending moments of the average patient were plotted as vector components in the neck coordinate system of a left hip implant (Figure 3). Slow and fast walking activities had components almost identical to normal walking and were therefore excluded for a clearer presentation of the graphs. The one-leg-standing activity was also excluded from the graphs for a better presentation of the other graphs as its numerous irregular fluctuations would deface the other graphs. All activities had a three dimensional dynamic behaviour. The *x*’ component of the bending moments dominated the *y*’ component and was positive during all activity cycles (i.e., no change in direction, Figure 3d,e). No single component of the frictional moments dominated the other two; and thus, ignoring any of these components for simplification should be justified. The frictional moment components of sit-to-stand, stand-to-sit, and knee bending activities had sharper changes of direction in comparison with normal walking, stair up, and stair down activities. There was no *z*’ component for the bending moment because the contact force passes through the centre point of the head located on the *z*’ axis of the neck coordinate system. These moment components are provided in the Cartesian system so that future finite element analysis (FEA) and experimental studies can use both magnitudes and directions over the activity cycle in a three dimensional manner.

**Figure 4:**
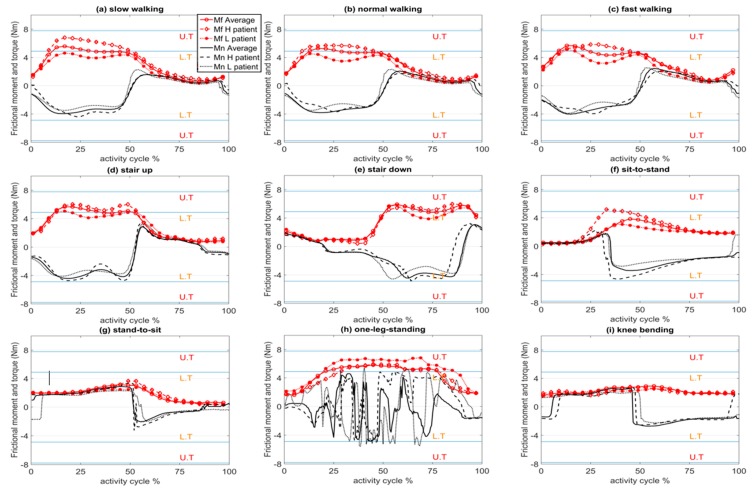
Frictional moment (*M*_f_) and torque at the head-neck interface (*M*_n_) for one cycle of each activity, for the average, heaviest (H), and lightest (L) patients. In the graphs, U.T and L.T indicate the upper and lower thresholds for depassivation, respectively. (**a**) Slow walking; (**b**) normal walking; (**c**) fast walking; (**d**) stair up; (**e**) stair down; (**f**) sit-to-stand; (**g**) stand-to-sit; (**h**) one leg standing and (**i**) knee bending.

Variations in the magnitude of the frictional moment vector ($\vec{M_{f}}$) were plotted against the activity cycle for the lightest, heaviest, and average patient for each physical activity (Figure 4). Both the magnitude and sign (direction) of the torque component of the frictional moment vector about the neck axis ($\vec{M_{n}}$) are shown in Figure 4 to illustrate the change of direction and variations in magnitude. To provide insight into fretting-corrosion initiation at the head-neck interface as a consequence of frictional torque, a lower threshold torque (L.T = 4.97 Nm) and an upper threshold torque (U.T = 7.78 Nm) for depassivation of the material surface are presented in Figure 4. These thresholds are the upper bounds of torque initiating corrosion at the head-neck interface, reported by Jauch et al. [17] for 4.5 kN and 6 kN assembled implants, respectively. It is acknowledged that these frictional torque thresholds are indicators of depassivation for the examined head-neck interface with its specific geometry (12/14 taper connection) and material characteristics. In general, fretting is a function of the stress field and micro-motions induced by the loads. The stress field and micro-motions themselves are functions of geometry [27]. Given that this taper geometry (12/14) is a common geometry for trunnion designs [28,29,30], the depassivation torque thresholds referenced in this work could provide a guide for this common type of head-neck taper design. To generalize the load related threshold, the stress field as a geometry independent indicator for depassivation should be available, so that it can be extended to all other geometries.

**Figure 5:**
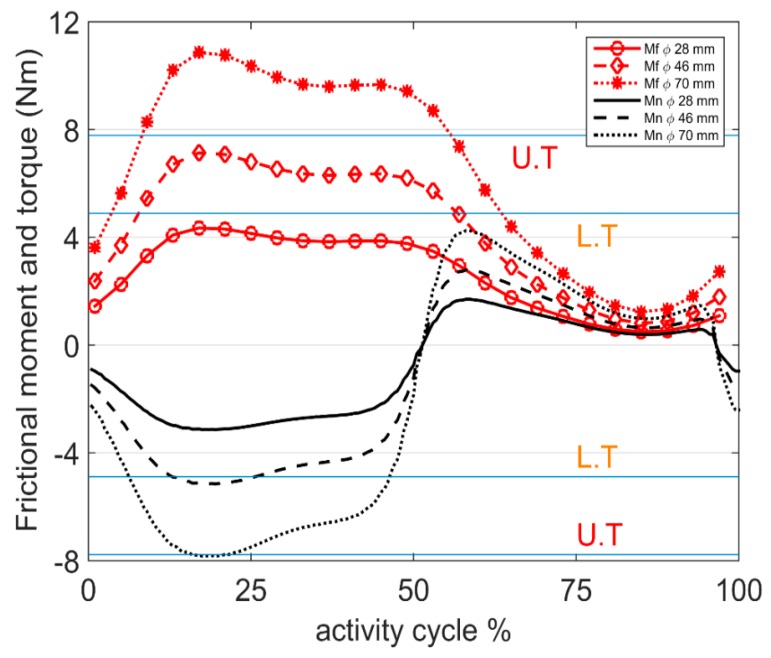
Frictional moment (*M*_f_) and torque at the head–neck interface (*M*_n_) for one cycle of normal walking, for the average patient, for different head diameters of 28, 46, and 70 mm. U.T = upper threshold for depassivation and L.T = lower threshold for depassivation.

It can be seen from Figure 4 that, in most simulated activities, the maximum and/or minimum torque values were near the L.T threshold. The frictional moments increased linearly with head size. If the bearing diameter is increased (Figure 5) to 70 mm, the torque exceeded the U.T threshold for walking. Recent studies have reported a direct relationship between increased bending moment at the head neck junction and increased oxide layer depassivation current [8]. However, threshold bending moments for depassivation have not been reported.

The maximum magnitude of the bending moments, frictional moments, and torques over the cycle of each activity is listed in Table 1 for the lightest, heaviest, and average patients. These maximum magnitudes ranged from 7 to 21.6 Nm for the bending moment, 2.6 to 7.1 Nm for the frictional moment, and 2.3 to 5.7 Nm for the torque acting on the head-neck interface. Generally, knee bending and stand-to-sit activities had the lowest maximum magnitudes and one-leg-standing caused the highest magnitudes of bending moment, frictional moment, and torque. The almost simultaneous occurrence of the maximum magnitudes of *M*_b_, *M*_f_, *M*_n_ and contact force P can be seen in Table 1 where the times of maximum moments, torque, and contact force ($t_{M_{b}},\;t_{M_{f}},\;t_{M_{n}},$ and $t_{P}$) in different patients and activities are approximately the same in 14 out of the 27 simulated cases. $t_{M_{b}}$ was found to be more similar to $t_{P}$ which is due to the direct relationship between the bending moment and contact force, regardless of the presence or absence of relative rotation. The same was not true for the frictional moment due to the dependency of the frictional moment on the occurrence and direction of the relative rotation. Depending on the activity, the torque at the head-neck interface had a number of changes in its direction. The number of changes of direction ranged from 1 to 4, with most cases changing direction twice over the activity cycle. However, one-leg-standing experienced 12–15 changes in the direction of the torque. In most cases, frictional torque appeared in both the positive and negative direction of the neck axis.

## 4. Discussion

This study determined the variations in the bending moment, frictional moment, and torque at the head-neck interface of a metal-on-metal bearing couple for different activities of daily living. The obtained moment and torque profiles can be employed in in vitro tests and computer simulations. 

As can be seen in Figure 3, stair up and normal walking activities had similar trends of variation in their frictional moment components, especially in *x*’ and *z*’. This could be because of similarities in their dynamics, as presented in Figure 2b,d. Moment components of the normal walking activity were almost in a range that covers the other activities. This suggests that in vitro tests based on a realistic walking cycle, (the most frequent daily activity [31]), may be an acceptable representation of the other daily activities in terms of the range of frictional moments. However, the contribution of sharp changes of frictional moment in some activities (sit-to-stand, stand-to-sit, and knee bending) should be evaluated before this simplification is implemented.

The flexion/extension degree of freedom (Figure 2) was dominant in determining the direction of the frictional moment vector ($\vec{M_{f}}$), especially the *x*’ and *z*’ components. The instants of switching between flexion and extension (peaks of the flexion graph in Figure 2) coincide with the change of direction of *M*_f*x*’_ and *M*_f*z*’_ (intersecting with 0 Nm in Figure 3a,c). Considering the small version/anti-version angle (usually in the range of ±10 degrees), the neck axis (*z*’-Figure 3f) receives a larger projection from the flexion-extension frictional moment (Figure 1). Consequently, changes in the direction of the frictional torque (intersecting with 0 Nm in Figure 4) also coincide with the peaks of the flexion—extension angles.

Among the studied activities, normal walking resulted in the smallest range (minimum to maximum peak) of frictional torque about the neck axis (6.1 Nm). An overall consistency can be seen in both the frictional moment and torque trends and, to some extent, their magnitudes during the three activities of slow, normal, and fast walking for all the studied patients. One-leg-standing caused the largest range (11 Nm) of frictional torque about the neck axis. This could result from un-interrupted contact force due to the body weight and muscle contraction forces on one leg combined with small flexion and extension “stabilising” movements leading to high positive and negative torques. Several changes in flexion and extension for stabilising could explain the high number of changes of the frictional moment direction and its torque component (12–15 times) during one-leg-standing. 

Based on the reported thresholds for critical torques initiating fretting corrosion at the head-neck interface (Figure 4), frictional torques in most activities were near the lower threshold. Disruption of the protective passive layer formed on the metal surface at the head-neck interface (due to the repetitively applied contact forces) may be intensified when assisted by the frictional moments. Given that frictional moments are thought to contribute to fretting-corrosion at the head-neck interface [8,17], experimental and numerical studies should be performed to better understand if simplified uni-axial force testing protocols represent realistic, six degree of freedom fretting-corrosion conditions. 

Frictional moments and torques were proportional to the head diameter of the implant. The relationship was linear, thus, one may scale the results of this study by the ratio of head diameters to estimate the moment and torque trends for other head sizes during the studied activities. For the 46 mm head, the torque about the head-neck interface exceeded the lower threshold for depassivation during the stance phase of walking. In the case of the 70 mm head, the torque exceeded the upper threshold for depassivation during the stance phase and the lower threshold during the swing phase. This suggests that with increasing head diameter, increased torsional loads at the head-neck interface may increase the potential for fretting-corrosion failure in the taper junction [30]. 

The bending moments conformed to the contact forces in all the activities to a high extent. The unidirectional bending moment about *x*’ contributes to both the normal and tangential forces of the contact area. The fretting regime of a contact area is a function of normal and tangential forces and may vary from elastic region, to partial slip and gross slip regions [27]. Panagiotidou et al. [8] reported that with increasing bending moment, the maximum fretting current (indicator of the oxide layer depassivation) increases and then decreases for a CoCr/Ti combination. For CoCr/CoCr and ceramic/CoCr head-neck combinations, the maximum fretting current increases with increasing bending moment. Donaldson et al. [32] reported that for one cycle of walking activity, a 10 mm increase in the head offset (which results in a linearly proportional increase in the bending moment) increases the micro-motions by 5 μm. The predicted moments in this study may be scaled with respect to the offset and bearing radius to calculate the bending moments and frictional moments, respectively.

Overall, the results suggest that six degree of freedom loading may be required to adequately simulate the mechanical environment of total hip replacement implants. The observed synchronicity of the maximum values of frictional moment vector ($\vec{M_{f}}$), torque vector at the head-neck interface ($\vec{M_{n}}$), and contact force vector ($\vec{P}$) suggests that applying a uni-directional contact force in the dominant direction (*z* axis), together with a bi-directional moment, may be an acceptable simplification of the in vivo mechanical environment of the implant. Given the significance of the frictional moment and torque in terms of both magnitude and direction, simplified mechanical tests of fretting-corrosion at the head-neck interface which apply only uni-directional contact forces [33,34] may considerably underestimate the severity of the mechanical environment by neglecting the depassivation effect of frictional torque on the contacting materials of the head and neck. The frictional torques estimated in this study were within the range reported to initiate depassivation [17]. Panagiotidou [8] recently reported an experimental method of applying coupled uni-directional torque and uni-directional contact force at the head-neck interface. This approach is a more reasonable testing method with two degrees of freedom in loading. However, it still neglects the multi-directional oscillatory nature of the frictional moment and may underestimate the severity of the in vivo mechanical environment; and consequently, overestimate the service longevity of the implant taper junction. 

## 5. Conclusions

Bending and frictional moments induced at the head-cup bearing, and frictional torques at the head-neck interface were determined for a metal-on-metal bearing couple during nine different daily activities. Frictional moments had a three dimensional nature, varying in both magnitude (maximum values ranging 2.6 to 7.1 Nm) and orientation (up to 15 changes of direction) over the activity cycles. The range of frictional moment components for walking covers the range of frictional moments in most activities except knee bending. However, the pattern of variation in frictional moment components for walking did not resemble the other activities. Calculation of frictional moments for different head sizes showed that frictional moment is linearly proportional to the head diameter. In most cases, frictional moment components in the lateromedial direction had the highest magnitude.

## Figures and Tables

**Table 1:** Maximum magnitude of bending moment (*M*_b_, Nm), frictional moment (*M*_f_, Nm), torque at the head-neck interface (*M*_n_, Nm), contact force (*P*, N) and their corresponding times (percent of activity cycle), number of changes in direction of the torque at the head-neck interface (#CD); for the lightest (L), heaviest (H), and average (Avg) patient.

Activity		*M*_b_	*M*_f_ (Nm)	*M*_n_ (Nm)	*P* (N)	$t_{M_{b}}$ (%)	$t_{M_{f}}$ (%)	$t_{M_{n}}$ (%)	*t_P_* (%)	#CD
**Slow Walking**	**L**	12.6	4.6	3.5	2066	17.0	17.0	17.0	17.0	2
	**H**	15.5	6.8	4.4	2133	17.0	17.0	23.5	16.5	3
	**Avg**	13.8	5.6	4.0	2057	16.0	15.5	16.0	15.0	2
**Normal Walking**	**L**	12.7	5.7	3.5	2030	13.5	14.0	14.0	14.0	2
	**H**	12.8	5.7	3.7	1769	21.0	22.5	25.5	19.0	3
	**Avg**	12.9	5.3	3.8	2285	17.5	17.5	19.5	17.5	2
**Fast Walking**	**L**	14.5	5.2	3.9	2330	13.0	13.0	13.0	13.0	2
	**H**	13.0	5.9	4.0	1998	26.5	26.5	26.0	26.5	3
	**Avg**	14.3	5.7	4.0	2456	14.0	14.5	15.0	14.0	2
**Stair Up**	**L**	14.6	5.1	4.1	2278	16.0	16.5	15.5	16.0	2
	**H**	14.1	6.0	4.1	1927	47.0	47.5	47.0	47.5	2
	**Avg**	13.7	5.7	4.7	2466	18.5	18.5	18.5	18.5	2
**Stair Down**	**L**	16.2	5.9	4.4	2626	54.5	55.0	55.0	54.5	2
	**H**	13.9	6.0	4.6	2212	89.0	85.0	64.0	89.5	2
	**Avg**	15.1	3.0	4.8	2553	56.0	56.0	62.0	56.0	2
**Sit-to-Stand**	**L**	9.5	3.1	2.8	1678	41.0	41.0	41.0	41.0	2
	**H**	11.7	5.3	4.7	2036	37.5	34.0	38.0	38.0	1
	**Avg**	9.9	3.8	3.4	1866	45.0	45.0	45.0	45.0	1
**Stand-to-Sit**	**L**	7.6	2.8	2.3	1266	45.5	55.5	44.0	45.0	2
	**H**	8.9	3.7	3.3	1504	49.5	53.0	50.0	49.5	4
	**Avg**	8.4	3.2	2.9	1529	45.0	51.5	45.0	45.5	4
**One-Leg-Standing**	**L**	21.6	7.1	5.7	3616	39.0	39.0	38.5	39.0	13
	**H**	14.5	6.0	4.9	2185	46.5	46.0	46.5	46.5	12
	**Avg**	14.5	5.8	4.8	2270	46.5	46.5	43.5	46.5	15
**Knee Bending**	**L**	7.6	2.6	2.3	1445	58.0	50.0	58.0	58.0	1
	**H**	7.1	3.0	2.7	1150	46.0	48.0	45.0	46.0	3
	**Avg**	7.7	3.0	2.7	1406	55.5	46.0	54.5	57.0	2

## References

[1] Preuss R., Haeussler K.L., Flohr M., Streicher R.M. (2012). Fretting corrosion and trunnion wear—Is it also a problem for sleeved ceramic heads?. Semin. Arthroplast..

[2] Fallahnezhad K., Farhoudi H., Oskouei R.H., Taylor M. (2016). Influence of geometry and materials on the axial and torsional strength of the head-neck taper junction in modular hip replacements: A finite element study. J. Mech. Behav. Biomed. Mater..

[3] Marks R. (2009). Body mass characteristics of hip osteoarthritis patients experiencing aseptic loosening, periprosthetic fractures, dislocation, and infections after total hip replacement. Clinicoecon. Outcomes Res..

[4] Grivas T.B., Savvidou O.D., Psarakis S.A., Bernard P.F., Triantafyllopoulos G., Kovanis I., Alexandropoulos P. (2007). Neck fracture of a cementless forged titanium alloy femoral stem following total hip arthroplasty: A case report and review of the literature. J. Med. Case Rep..

[5] Hua X., Li J., Wang L., Jin Z., Wilcox R., Fisher J. (2014). Contact mechanics of modular metal-on-polyethylene total hip replacement under adverse edge loading conditions. J. Biomech..

[6] Di Puccio F., Mattei L. (2015). Biotribology of artificial hip joints. World J. Orthop..

[7] Damm P., Dymke J., Ackermann R., Bender A., Graichen F., Halder A., Beier A., Bergmann G. (2013). Friction in total hip joint prosthesis measured in vivo during walking. PLoS ONE.

[8] Panagiotidou A., Meswania J., Osman K., Bolland B., Latham J., Skinner J., Haddad F.S., Hart A., Blunn G. (2015). The effect of frictional torque and bending moment on corrosion at the taper interface: An in vitro study. Bone Jt. J..

[9] Bergmann G., Graichen F., Rohlmann A., Bender A., Heinlein B., Duda G.N., Heller M.O., Morlock M.M. (2010). Realistic loads for testing hip implants. Biomed. Mater. Eng..

[10] Bishop N.E., Hothan A., Morlock M.M. (2013). High friction moments in large hard-on-hard hip replacement bearings in conditions of poor lubrication. J. Orthop. Res..

[11] Bishop N.E., Waldow F., Morlock M.M. (2008). Friction moments of large metal-on-metal hip joint bearings and other modern designs. Med. Eng. Phys..

[12] Oskouei R.H., Fallahnezhad K., Kuppusami S. (2016). An investigation on the wear resistance and fatigue behaviour of Ti–6Al–4V notched members coated with hydroxyapatite coatings. Materials.

[13] Swaminathan V., Gilbert J.L. (2012). Fretting corrosion of CoCrMo and Ti6Al4V interfaces. Biomaterials.

[14] English T.A., Kilvington M. (1979). In vivo records of hip loads using a femoral implant with telemetric output (a prelimary report). J. Biomed. Eng..

[15] Bergmann G., Graichen F., Rohlmann A. (1993). Hip joint loading during walking and running, measured in two patients. J. Biomech..

[16] Bergmann G., Deuretzbacher G., Heller M., Graichen F., Rohlmann A., Strauss J., Duda G.N. (2001). Hip contact forces and gait patterns from routine activities. J. Biomech..

[17] Jauch S.Y., Coles L.G., Ng L.V., Miles A.W., Gill H.S. (2014). Low torque levels can initiate a removal of the passivation layer and cause fretting in modular hip stems. Med. Eng. Phys..

[18] California Joint Replacement Registry (CJRR) (2013). CJRR Annual Report: Hip and Knee Replacements in Canada.

[19] Li S., Chien S., Brånemark P.I. (1999). Heat shock-induced necrosis and apoptosis in osteoblasts. J. Orthop. Res..

[20] Caouette C., Bureau M.N., Vendittoli P.A., Lavigne M., Nuño N. (2015). Influence of the stem fixation scenario on load transfer in a hip resurfacing arthroplasty with a biomimetic stem. J. Mech. Behav. Biomed. Mater..

[21] Avval P.T., Samiezadeh S., Klika V., Bougherara H. (2015). Investigating stress shielding spanned by biomimetic polymer-composite vs. metallic hip stem: A computational study using mechano-biochemical model. J. Mech. Behav. Biomed. Mater..

[22] Zhang T., Harrison N.M., McDonnell P.F., McHugh P.E., Leen S.B. (2013). A finite element methodology for wear–fatigue analysis for modular hip implants. Tribol. Int..

[23] Farhoudi H., Oskouei R.H., Jones C.F., Taylor M. (2015). A novel analytical approach for determining the frictional moments and torques acting on modular femoral components in total hip replacements. J. Biomech..

[24] Liu F., Jin Z.M., Hirt F., Rieker C., Roberts P., Grigoris P. (2006). Transient elastohydrodynamic lubrication analysis of metal-on-metal hip implant under simulated walking conditions. J. Biomech..

[25] Meng Q.E., Liu F., Fisher J., Jin Z.M. (2011). Transient elastohydrodynamic lubrication analysis of a novel metal-on-metal hip prosthesis with a non-spherical femoral bearing surface. Proc. Inst. Mech. Eng..

[26] Meng Q., Wang J., Yang P., Jin Z., Fisher J. (2015). The lubrication performance of the ceramic-on-ceramic hip implant under starved conditions. J. Mech. Behav. Biomed. Mater..

[27] Neu R.W. (2011). Progress in standardization of fretting fatigue terminology and testing. Tribol. Int..

[28] Bolland B.J., Culliford D.J., Langton D.J., Millington J.P., Arden N.K., Latham J.M. (2011). High failure rates with a large-diameter hybrid metal-on-metal total hip replacement: Clinical, radiological and retrieval analysis. J. Bone Jt. Surg. Br..

[29] Nassif N.A., Nawabi D.H., Stoner K., Elpers M., Wright T., Padgett D.E. (2014). Taper Design Affects Failure of Large-head Metal-on-metal Total Hip Replacements. Clin. Orthop. Relat. Res..

[30] Panagiotidou A., Bolland B., Meswania J., Skinner J., Haddad F., Hart A., Blunn G. (2013). Effect of increased frictional torque on the fretting corrosion behaviour of the large diameter femoral head. Bone Jt. J..

[31] Morlock M., Schneider E., Bluhm A., Vollmer M., Bergmann G., Müller V., Honl M. (2001). Duration and frequency of every day activities in total hip patients. J. Biomech..

[32] Donaldson F.E., Coburn J.C., Siegel K.L. (2014). Total hip arthroplasty head–neck contact mechanics: A stochastic investigation of key parameters. J. Biomech..

[33] Gilbert J.L., Mehta M., Pinder B. (2009). Fretting crevice corrosion of stainless steel stem–CoCr femoral head connections: Comparisons of materials, initial moisture, and offset length. J. Biomed. Mater. Res. B Appl. Biomater..

[34] Goldberg J.R., Gilbert J.L. (2003). In vitro corrosion testing of modular hip tapers. J. Biomed. Mater. Res. B Appl. Biomater..

